# Proteomic Analysis of ISGylation in Immortalized Porcine Alveolar Macrophage Cell Lines Induced by Type I Interferon

**DOI:** 10.3390/vaccines9020164

**Published:** 2021-02-17

**Authors:** Chengbo Zhu, Jingrui Li, Chaonan Tian, Mengmeng Qin, Zhenni Wang, Bingjun Shi, Guanggang Qu, Chunyan Wu, Yuchen Nan

**Affiliations:** 1 Department of Preventive Veterinary Medicine, College of Veterinary Medicine, Northwest A&F University, Yangling 712100, China; chengbozhu@nwafu.edu.cn (C.Z.); ttttian121@163.com (C.T.); qinmengmeng111@163.com (M.Q.); wzn02060207@163.com (Z.W.); 13333482298@163.com (B.S.); 2 State Key Laboratory of Plant Physiology and Biochemistry, College of Biological Sciences, China Agricultural University, Beijing 100193, China; jrli@cau.edu.cn; 3 Shaanxi Animal Disease Control Center, Xi’an 710000, China; 4 Binzhou Animal Science and Veterinary Medicine Academy, Binzhou 256600, China

**Keywords:** interferons, interferons-stimulated genes (ISGs), ISG15, ISGylation, ubiquitination

## Abstract

Interferon-stimulated gene product 15 (ISG15), a ubiquitin-like molecule, can be conjugated to protein substrates through a reversible process known as ISGylation. ISG15 and ISGylation are both strongly upregulated by type I interferons and play putative key roles in host innate immunity against viral infection. However, the function of ISGylation and identities of ISGylation substrates are largely unknown. Here, a novel monoclonal antibody (Mab) that specifically recognizes porcine ISG15 (pISG15) was employed to capture ISG15-conjugated proteins from IFNs-stimulated porcine cell lysates. Next, Mab-captured conjugates were analyzed using proteomics-based tools to identify potential ISGylation protein targets in order to elucidate the roles of ISG15 and ISGylation in porcine cells. Subsequently, 190 putative ISGylation sites were detected within 98 identified ISGylation candidates; several candidates contained more than one ISGylation-modifiable lysine residue, including pISG15 itself. Motif enrichment analysis of confirmed ISGylation sites demonstrated a moderate bias towards certain sites with specific upstream amino acid residues. Meanwhile, results of Gene Ontology (GO)-based annotation and functional enrichment and protein-protein interaction (PPI) network analyses of porcine ISG15-conjugated substrate proteins indicated that these substrates were mainly associated with the host metabolism, especially nucleotide metabolic pathways that ultimately may participate in cellular antiviral defenses. Notably, several ISGs (MX1, IFIT1, OAS1, ISG15 and putative ISG15 E3 ligase Herc6) were also identified as putative ISGylation substrates within a regulatory loop involving ISGylation of ISGs themselves. Taken together, proteomics analysis of porcine ISGylation substrates revealed putative functional roles of ISG15 and novel host ISGylation targets that may ultimately be involved in cellular antiviral responses.

## 1. Introduction

Interferons (IFNs), a group of secreted cytokines that play key roles in host antiviral immunity, are defined by their abilities to inhibit virus replication in vitro and in vivo. So far, three types of IFNs (type I, II and III) have been characterized [1]. Type I IFNs, the most well-known IFN type, constitute the largest of the three IFN families [2] and include IFN-α, IFN-β, IFN-ε, IFN-κ and IFN-ω [3,4]. All type I IFNs bind to a ubiquitously expressed heterodimeric receptor complex composed of IFN-α receptors IFNAR1 and IFNAR2 to activate Janus kinase and signal transducer and activator of transcription (JAK/STAT) pathways. The Type II IFN family, which is solely comprised of IFN-γ, is distinct from type I IFNs, due to its different receptors and distinct chromosomal location [2,5]. IFN-γ signal transduction depends on participation of a broadly expressed IFN-γ receptor complex (IFNGR) [6,7,8]. IFN-γ plays a central role in establishing cellular immunity, while also inducing expression of a specific group of antiviral genes which apparently also respond to type I IFNs as well [9,10]. The Type III IFN family, the most recently discovered family, is comprised of IFN-λs 1 through 4 [5,11]. IFN-λ signaling is mediated by a heterodimeric receptor composed of IFNLR1 (or IL-28R) and IL-10R2 that is quite distinct from receptors triggered by type I and type II IFNs. Nevertheless, IFN-λ activates the same downstream pathways as those triggered by type I IFNs and induces expression of similar downstream genes. Thus, all IFNs stimulate cells via binding of their corresponding receptors to trigger JAK/STAT pathway activation [12] that subsequently evokes a sequential cascade of events leading to increased expression of IFN-stimulated genes (ISGs) [13]. Generally, more than 300 ISGs have been shown to be upregulated by type I IFNs [14]. 

IFN-stimulated gene product 15 (ISG15) was identified soon after the discovery of ubiquitin and is recognized as a ubiquitin homologue [15]. Mature human ISG15 protein is shorter than its precursor, due to the removal of 8 C-terminal amino acids, and retains a shared C-terminal amino acid motif LRLRGG with mature ubiquitin [16], which is capable of undergoing conjugation with protein substrates. However, unlike other ubiquitin homologues, such as small ubiquitin-like modifier (SUMO) or neural precursor cell-expressed developmentally down-regulated gene 8 (Nedd8), ISG15 orthologues have not been detected in yeast, insects, plants or other lower organisms, implying that ISG15 performs a highly specialized function in vertebrates [17]. Moreover, sequence analysis has demonstrated that ISG15 is not conserved among mammalian species, suggesting that ISG15 plays distinct species-specific antiviral roles in different hosts [18].

As a ubiquitin-like molecule, ISG15 can be conjugated to protein substrates through a reversible process known as ISGylation [15]. While it has been demonstrated that protein ISGylation does not lead to substrate degradation, as occurs after K48-linked ubiquitination, no universal role of protein ISGylation has been identified. Indeed, it is still unclear whether ISGylated proteins share a common fate similar to ubiquitination or if fates of individual ISGylated proteins vary [19,20]. Nevertheless, ISG15 expression is strongly upregulated after IFN treatment, supporting an important role for ISG15 in the host innate immune response. Moreover, it has been reported that ISG15-deficient mice exhibit increased susceptibility to influenza A and B viruses, Sindbis virus and HSV-1 [21,22]. Early studies using proteomics-based approaches have also identified 76 total ISGylation candidates in IFNs-treated mouse and human cells, with only 21 of these putative targets overlapping between species [23], evidence that ISG15-conjugated substrates may be species-specific as well. However, natural substrates of porcine ISG15 have not yet been investigated, due to the lack of antibodies that specifically bind to porcine ISG15.

In our previous report, we described development of a novel monoclonal antibody (Mab) that specifically recognized endogenous porcine ISG15 (pISG15) produced by IFNs-stimulated porcine cells but did not cross-react with ISG15 from other species [24]. Here we utilized this Mab to capture pISG15-conjugated substrates from IFNs-treated porcine cells then conducted proteomics analyses of potential targets of porcine ISGylation to reveal antiviral roles played by ISG15-conjugated proteins in porcine cells. Our data here demonstrated that ISG15 and ISG15-conjugated targets were mainly associated with host metabolic pathways, while several antiviral proteins, such as MX1, IFIT1, OAS1 and ISG15, were also identified as ISGylation candidates. In summary, our data reveal a novel function of ISG15 in porcine cells that is tied to cellular antiviral responses. 

## 2. Material and Methods

### 2.1. Cells and Chemicals 

Immortalized porcine alveolar macrophage cell line 3D4/21 ATCC^®^ CRL-2843 cells were obtained from the American Type Culture Collection (ATCC, Manassas, VA, USA) and maintained in Dulbecco’s Modified Eagle Medium (DMEM; Biological Industries, Israel) supplemented with 10% FBS (Biological Industries). Recombinant porcine IFN-α (pIFN-α) was purchased from R&D Systems (Minneapolis, MN, USA) and used to treat CRL-2843 cells at a concentration of 10 ng/mL. 

### 2.2. Western Blot (WB) Analysis

Whole cell lysates of CRL-2843 cells with or without pIFN-α treatment were prepared by addition to cells of 1× Laemmli sample buffer (Bio-Rad Laboratories, Hercules, CA, USA). Lysate proteins were separated by sodium dodecyl sulfate-polyacrylamide gel electrophoresis (SDS-PAGE) [25] then separated proteins were transferred to PVDF membranes as previously reported [26]. Membranes were probed with homemade anti-porcine ISG15 (pISG15) Mab-3D5E6 or anti-β-tubulin Mab (Transgene, Beijing, China). Specific binding interactions between antibodies and corresponding targets were detected using HRP-conjugated goat anti-mouse IgG (H+L) (Thermo Fisher Scientific, Waltham, MA, USA) and visualized using enhanced chemiluminescent (ECL) substrate-based detection (Bio-Rad Laboratories). Chemiluminescence signal acquisition was conducted using a ChemiDoc MP Imaging System (Bio-Rad Laboratories) and data were analyzed using ImageLab software (Version 5.1, Bio-Rad Laboratories). 

### 2.3. Immuno-Precipitation and Enrichment of ISG15-Conjugated Proteins

CRL-2843 cells were treated with pIFN-α for 24 hours then were lysed using RIPA buffer (Thermo Fisher Scientific) supplemented with protease inhibitor cocktail (Sigma-Aldrich, St. Louis, MO, USA) and de-ubiquitination inhibitor N-ethylmaleimide (NEM; Sigma-Aldrich) at a final concentration of 50 µM to block de-ISGylation process mediated by cellular deubiquitinating enzyme during immuno-precipitation. Each lysate was clarified by centrifugation at 14,000× *g* for five minutes at 4 °C. Next, 10 μg of anti-porcine ISG15 Mab-3D5E6 was added to each cell lysate, then treated lysates were incubated at 4 °C for three hours to capture free ISG15 and ISG15-conjugated proteins. Next, 25 μL of protein G agarose beads (Genscript, Nanjing, China) was then added to each cell lysate and each mixture was incubated for another hour to permit immune complex formation. The supernatant was removed by centrifugation at 10,000× *g* at 4 °C then the immunoprecipitated (IP) pellet was washed three times with RIPA buffer supplemented with protease inhibitor cocktail and NEM before harvesting protein G beads with 1× Laemmli sample buffer. Next, Laemmli sample buffer harvested beads were boiled for ten minutes followed by cooling on ice for an additional five minutes before centrifugation under 14,000× *g* for five minutes. The supernatant contained IP proteins were subjected to Western blot (WB) analysis to assess their abilities to bind to rabbit anti-porcine ISG15 polyclonal antibodies in order to verify their ISG15 conjugation status.

### 2.4. Mass Spectrometry-Based Peptides Analysis 

In order to enrich pISG15-conjugated proteins for mass spectrometry, an immunoaffinity column containing pISG15-binding Mab-3D5E6 was prepared to avoid contamination with mouse Mab associated with IP-based enrichment. Specifically, CNBr-activated Sepharose 4B resin (GE Healthcare, Chicago, IL, USA) was washed with cold 1 mM HCl and conjugated with Mab-3D5E6 following the manufacturer’s instructions, at a ratio of 1 mL resin to 8 mg Mab-3D5E6. Meanwhile, a sample containing a total of 1 × 10^8^ pIFN-α-treated CRL-2843 cells was treated with RIPA lysis buffer (Thermo Fisher Scientific) supplemented with protease inhibitor cocktail (Sigma-Aldrich) and NEM (Sigma-Aldrich) at a final concentration of 50 µM. Next, 100 μL of Mab-3D5E6-conjugated Sepharose 4B resin beads were added to each cell lysates followed by incubation at 4 °C for three hours to capture free pISG15 and pISG15-conjugated substrates. Supernatant was removed by centrifugation at 10,000× *g* at 4 °C then the Sepharose 4B beads were washed three times with RIPA buffer, supplemented with protease inhibitor cocktail and NEM, before adding 8 M urea to harvest proteins. Protein concentration determinations were conducted using a BCA kit according to the manufacturer’s instructions.

Mass spectrometry-based peptides analysis was conducted as previously described with modifications [27]. Briefly, for protein trypsin digestion, the protein solution was reduced via addition of 5 mM dithiothreitol followed by incubation for 30 min at 56 °C then proteins were alkylated by addition of 11 mM iodoacetamide followed by incubation for 15 min at room temperature in the dark. Treated protein samples were then diluted by adding 100 mM tetraethylammonium bromide (TEAB) to decrease the urea concentration to below 2 M. Finally, trypsin was added at a 1:50 trypsin-to-protein mass ratio for the first digestion overnight and 1:100 trypsin-to-protein mass ratio for a second 4-h digestion. Next, resulting tryptic peptides were dissolved in 0.1% formic acid (solvent A) then directly loaded onto a homemade reversed-phase analytical column (15-cm length, 75-μm i.d.). The gradient was increased from 6% to 23% solvent B (0.1% formic acid in 98% acetonitrile) over 26 min, increased from 23% to 35% in 8 min, then increased to 80% in 3 min and held at 80% for the last 3 min, all achieved using a constant flow rate of 400 nL/min using an EASY-nLC 1000 UPLC System. Peptides were subjected to nano-spray ionization followed by tandem mass spectrometry (MS/MS) in Q Exactive^TM^ Plus (Thermo Fisher Scientific) coupled online to the UPLC System. The electrospray voltage applied was 2.0 kV, m/z scan range was 350 to 1800 for the full scan and intact peptides were detected in the Orbitrap at a resolution of 70,000. Peptides were then selected for MS/MS using 28 as the normalized collision energy (NCE) setting and fragments were detected in the Orbitrap at a resolution of 17,500. The data-dependent procedure alternated between one MS scan followed by 20 MS/MS scans using a 15.0-s dynamic exclusion window, automatic gain control (AGC) set to 5E4 and fixed first mass set to 100 m/z. 

Resulting MS/MS data were processed using the Maxquant search engine (v.1.5.2.8) using the *Sus_scrofa*_9823_PR_20200506 database for peptide mapping. Since peptides containing putative ISG15-conjugation sites generated the same residues as ubiquitination after trypsin digestion (involving GG residues conjugated to lysine within peptides), tandem mass spectra were searched against a ubiquitination database concatenated to a reverse decoy database. Trypsin/P was specified as cleavage enzyme to allow for up to 2 missed cleavage sites. Mass tolerance for precursor ions was set to 20 ppm in the First search and 5 ppm in the Main search, while mass tolerance for fragment ions was set to 0.02 Da. Carbamidomethylation of Cys was specified as a fixed modification, while oxidation of Met was specified as a variable modification. The false discovery rate (FDR) threshold was adjusted to <1% and the minimum score for peptides was set to >40. 

### 2.5. Gene Ontology Annotation and Functional Enrichment

Gene Ontology (GO) annotation of all identified protein candidates was conducted using the UniProt-GOA database (https://www.ebi.ac.uk/GOA/ (accessed on 19 February 2021)). After converting identified protein IDs to their UniProt IDs, all candidates were annotated using GO analysis based on their UniProt IDs. If a candidate could not be annotated using the UniProt-GOA database, the InterProScan (http://www.ebi.ac.uk/interpro/ (accessed on 19 February 2021)) was used to annotate its GO function using a protein sequence alignment method. All candidates were classified based on GO annotation categories of biological process, cellular component and molecular function. Functional analyses were conducted using Clusters of Orthologous Groups of proteins (COG/KOG) based on the NCBI database for prokaryotes (COG database). COG/KOG functional classifications of all identified proteins were performed via database comparisons and analysis. After initial GO enrichment analysis classification of identified proteins into GO annotation categories (biological process, cellular compartment, molecular function), a two-tailed Fisher’s exact test was employed to test the enrichment of identified proteins against all proteins of the species database. GO results with corrected *p*-values < 0.05 were considered significant.

### 2.6. Protein-Protein Interaction Network

To investigate protein-protein interaction (PPI) networks associated with all identified proteins, accession numbers or sequences of all candidate proteins were searched against the STRING database (version 10.5). Only interactions between proteins belonging to searched data sets were selected, thereby excluding external candidates. STRING defines a metric designated as a “confidence score” to define interaction confidence; only interactions with confidence scores > 0.7 (high confidence) were fetched for use in identifying interaction networks using STRING that were then visualized using R package networkD3 module.

## 3. Result

### 3.1. Mab-3D5E6 Recognized Endogenous ISG15 Produced by Porcine Cells and Captured ISG15-Conjugated Proteins via Immunoprecipitation

**Figure 1:**
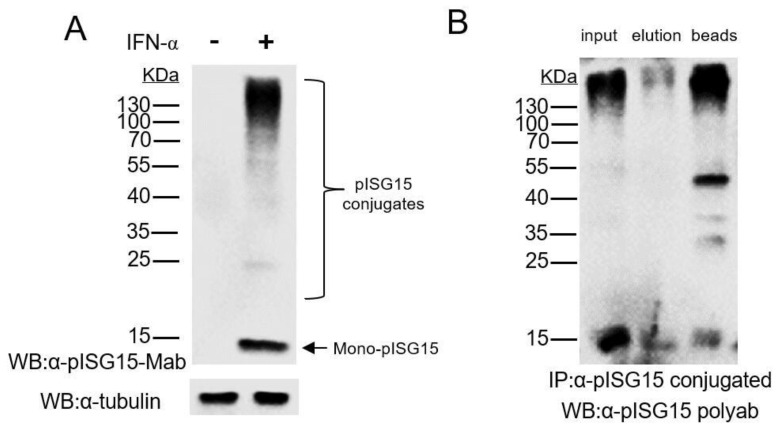
Enrichment of interferon-stimulated gene product 15 (ISG15)-conjugated proteins from porcine cells. (**A**) Porcine ISG15 (pISG15)-specific Mab-3D5E6 recognized endogenous pISG15 in porcine cells. CRL-2843 cells were treated with porcine IFN-α for 24 hours then harvested for SDS-PAGE and Western blotting using anti-pISG15 Mab-3D5E6. (**B**) Immuno-precipitation (IP) of free pISG15 and pISG15-conjugates: CRL-2843 cells were treated with porcine IFN-α then harvested using RIPA buffer for IP containing Mab-3D5E6; Western blotting was conducted to detect free pISG15 and ISG15-conjugates from supernatant and in precipitated immunocomplexes.

In our previous research, we generated a monoclonal antibody (Mab) against porcine ISG15 (pISG15) via immunization of mice with recombinant pISG15 expressed in *Escherichia coli* then screened surviving hybridoma clones for ISG15-binding specificity using ELISA [24]. After completion of all screening assays and sub-cloning procedures, hybridoma clone No. 3D5E6 (Mab-3D5E6) was selected and was shown, via immunofluorescence assay (IFA), to exhibit good binding reactivity to endogenous pISG15 produced by an IFN-stimulated porcine cell line. To determine whether Mab-3D5E6 could be used to capture ISG15-conjugated proteins produced by IFNs-stimulated porcine cells, we subsequently tested its binding reactivity to endogenous ISG15 and ISG15-conjugated cellular proteins produced by pIFN-α-stimulated CRL-2843 cells (an immortalized cell line derived from porcine alveolar macrophages) using WB analysis. Mab-3D5E6 was shown to strongly bind to ISG15 and ISG15-conjugated CRL-2843 cellular proteins (Figure 1A). Moreover, when used to capture ISG15-conjugated proteins from CRL-2843 cells, Mab-3D5E6 was shown to capture the majority of immunoprecipitated proteins, including both free ISG15 and ISG15-conjugated proteins, from cell lysates (Figure 1B). Thus, Mab-3D5E6 successfully captured free pISG15 and pISG15-conjugated cellular protein targets.

### 3.2. Mass Spectrometry Analysis of Porcine ISG15-Conjugated Proteins

To obtain the full spectrum of ISG15-conjugated proteins from CRL-2843 cells after IFNs treatment, Mab-3D5E6-captured proteins were subjected to trypsin digestion and mass spectrometry (MS) analysis. As demonstrated in Figure S1, QC analysis indicated that lengths of most peptides within trypsin-treated samples ranged from 7 to 20 amino acids (aa) residues, which matched prospective results obtained for enriched proteins processed via trypsin digestion and higher-energy C-trap dissociation (HCD). Ultimately, search results obtained using the porcine database (*Sus_scrofa*_9823_PR_20200506) predicted a total of 82,281 possible peptides within input sample proteins of which 262 peptides contained prospective ISG15-conjugation sites (GGR motifs within peptides) (Table 1). Within these candidate peptides, 190 modification sites were identified and mapped to 98 individual proteins (Table 1), suggesting that several putative ISGylation candidates contained more than one lysine residue for ISGylation. All identified peptide sequences, putative modification sites and protein descriptions are summarized in Table S1. Based on the limited number of ISGylation candidates, ISGylation does not appear to be as common as protein ubiquitination, a universal post-translation modification that can modify any protein. These observations are also consistent with the existence of a limited number of ISG15-specific E3 ligases, as described previously [28,29], as well as to the limited number of ISGylated proteins detected previously in human and mouse species [23,30].

**Figure 2:**
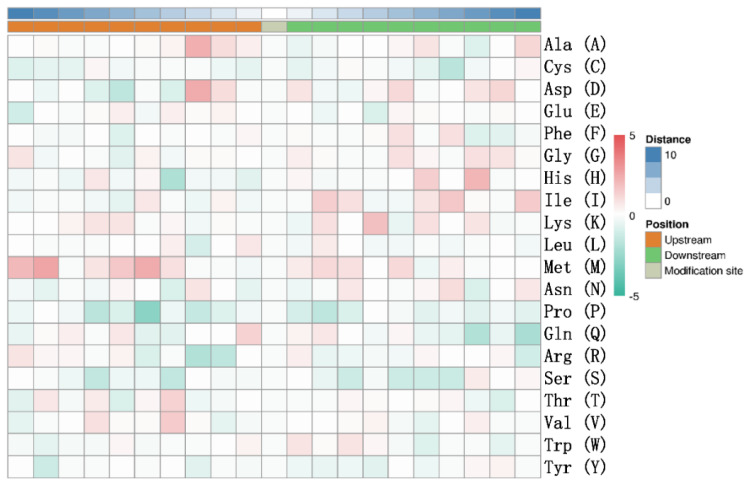
Motif heatmap of upstream and downstream amino acids of all ISGylation sites. Motifs of both upstream and downstream 10 amino acids of ISGylation sites from all identified peptides were analyzed for motif enrichment. The red color represents corresponding amino acids that were significantly enriched at indicated sites, while green color represents corresponding amino acids that were significantly reduced at indicated sites.

One factor limiting the number of ISGylated proteins may reflect a requirement for participation of sequence motifs surrounding protein ISGylation sites, whereby certain upstream or downstream sequence motifs may influence the likelihood of occurrence of ISGylation at a particular site. To investigate this possibility, a motif enrichment heatmap of upstream and downstream aa sequences of all identified ISGylation sites within candidate peptides was generated (Figure 2). Based on the motif heat map (Figure 2), no universal or conserved motif could be identified either upstream or downstream of putative ISGylation sites though it appears that sequences upstream of ISGylation sites were more likely to contain biased aa residues than downstream sequences; for example, the 9th and 5th aa residues upstream of ISGylation sites were moderately biased toward Met residues with the 5th aa residues less biased toward Pro residue. Moreover, while Thr and Val residues were preferentially observed at the 4th aa residue site upstream of the identified ISGylation site (Figure 2). Meanwhile, the Ala and Asp residues at the 3rd position upstream appears to be biased as well. Conversely, the His residue at position 8 downstream and Lys residue at position 4 downstream appears to be biased, respectively (Figure 2). However, whether these biased residues upstream or downstream of potential ISG15-conjugating sites imply a potential selection motif, which allows only limited substrates of total cellular proteins to be targeted by host ISGylation system, or if these biased residues were incidentally identified due to limited numbers of potential candidates available for motif analysis still requires further investigation.

### 3.3. Functional Categories of Porcine ISG15-Conjugated Proteins

**Figure 3:**
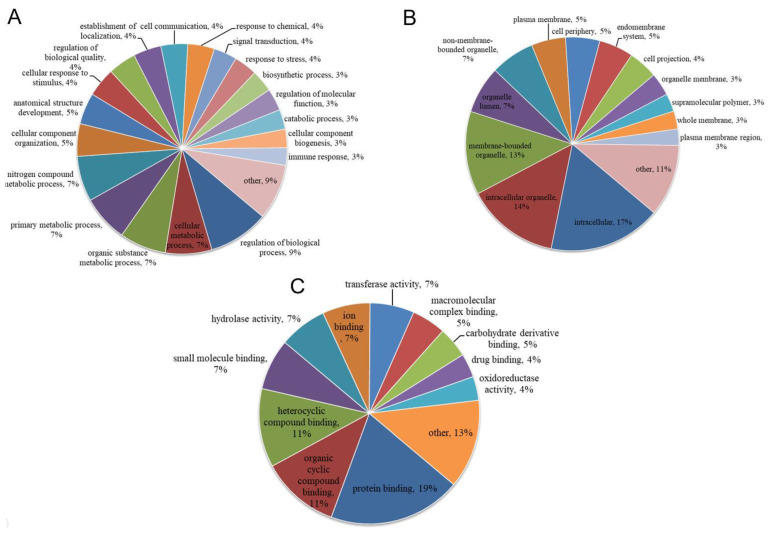
Statistical distribution chart of interferon-stimulated gene product 15 (ISG15)-conjugated proteins under each Gene Ontology (GO) category. (**A**) Distribution chart of identified proteins under the GO category of biological process. (**B**) Distribution chart of identified proteins under the GO category of cellular component, (**C**) Distribution chart of identified proteins under the GO category of molecular function.

To further investigate ISG15-conjugated proteins produced by IFNs-stimulated porcine cells, all identified proteins with putative ISGylation sites were subjected to further analysis using various methods. First, Gene Ontology (GO)-based annotation was conducted using the UniProt-GOA database to assign all identified proteins to three GO functional categories: biological process (Figure 3A and Table S2), cellular component (Figure 3B and Table S2) or molecular function (Figure 3C and Table S2). Based on category assignment, top terms were identified for each protein. The top five annotation terms for biological process-assigned ISGylated proteins were “regulation of biological process”, “cellular metabolic process”, “organic substance metabolic process”, “primary metabolic process” and “nitrogen compound metabolic process” (Figure 3A), which suggested that natural substrates of the ISG15-conjugation system in porcine cells were associated with metabolism. Although it is unclear whether ISGylation confers a “gain of function” or “loss of function” to these metabolism-related proteins, alterations of metabolic pathways would definitely affect virus replication, since biosynthesis of viral components is strictly host-dependent. By contrast, unpredictably only a limited number of immune-related genes (3% of total identified proteins) were targeted by the ISG15-conjugation system that included well-defined ISGs, such as MX1, IFIT1, OAS1 and ISG15 itself (Table S2). It is also notable that multiple ISGylation sites (K6, K8, K13, K29, K128 and K142) within ISG15 were identified (Table S2), which implies that ISG15 may form poly-ISG15 chains similar to polyubiquitin chains. Besides biological process analysis, subcellular locations of identified proteins undergoing ISG15-conjugation were analyzed as well (Figure 3B). In contrast to results of a previous report demonstrating that the human ISG15 conjugation system broadly targeted newly synthesized proteins [31], our data suggested that porcine ISG15-conjugated proteins were widely distributed among all subcellular locations (Figure 3B and Table S2). Moreover, molecular function annotation analyses of porcine ISG15-conjugates (Figure 3C) revealed five top assigned functions of “protein binding”, “organic cyclic compound binding”, “heterocyclic compound binding”, “small molecule binding” and “hydrolase activity” as further support for the concept that ISGylation targets substrates within host metabolic pathways.

**Figure 4:**
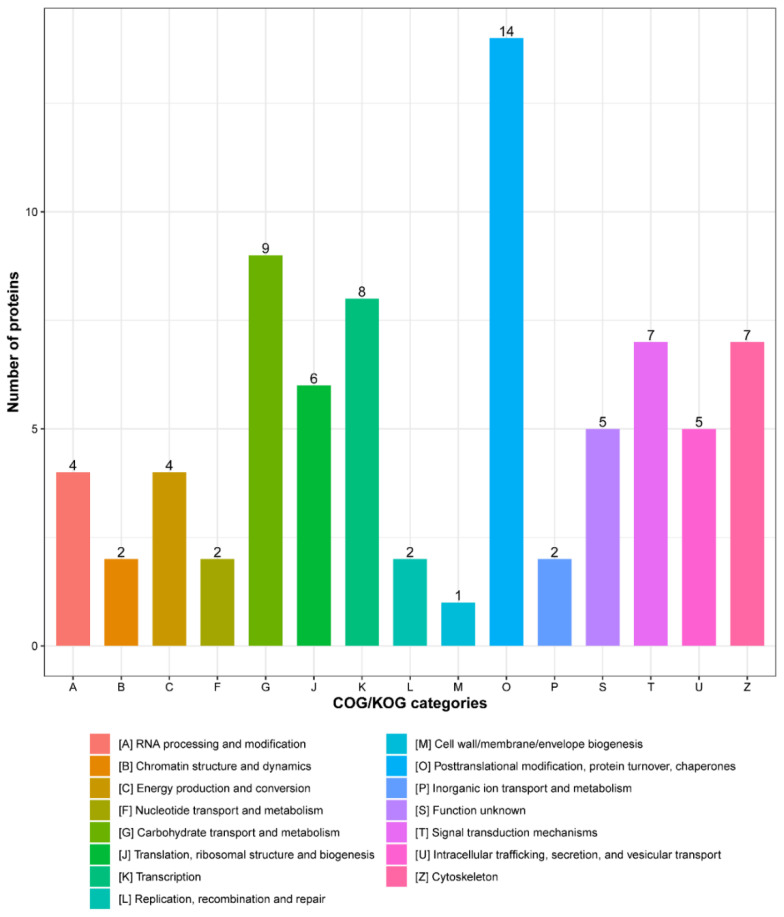
Clusters of Orthologous Groups (COG) functional classification of interferon-stimulated gene product 15 (ISG15)-conjugated proteins.

To further functionally classify detected ISG15-conjugated proteins, they were subjected to Clusters of Orthologous Groups (COG) analysis. Based on analysis results (Figure 4 and Table S3), the top 5 COG categories identified included “Posttranslational modification, protein turnover, chaperones”, “Carbohydrate transport and metabolism”, “Transcription”, “Cytoskeleton” and “Signal transduction mechanisms”. COG data aligned with Gene Ontology (GO) annotation results, while also pinpointing other pathways, such as “Transcription”, “Cytoskeleton” and “Signal transduction mechanisms” as associated with ISG15-conjugation system targets. Importantly, our results differed from results of a previous study that identified top functional categories for ISG15-conjugated substrates in human cells (HeLa cells) [30] of “RNA processing”, “Cytoskeleton organization/regulation”, “metabolism”, “Chromatin remodeling/pol II transcription”, “stress response” and “translation” [30]. Taken together, differences in the abovementioned results suggest that the porcine ISG15-conjugation system differs from the human ISG15 conjugation system in that the porcine system preferentially targets proteins of host metabolic pathways for ISGylation. Ultimately, these results indicate that alteration of host metabolism may be a primary mechanism underlying the ISG15 antiviral effect observed in porcine cells.

### 3.4. Functional Enrichment of Porcine ISG15-Conjugated Proteins

**Figure 5:**
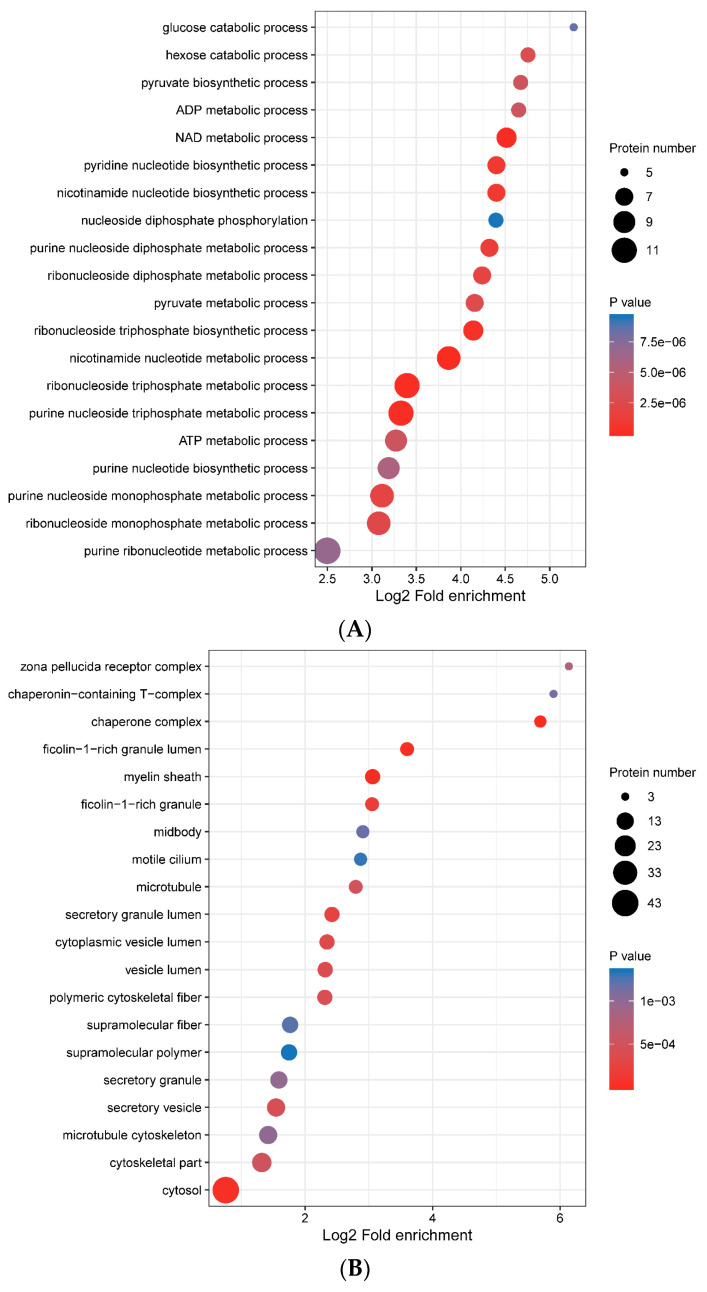

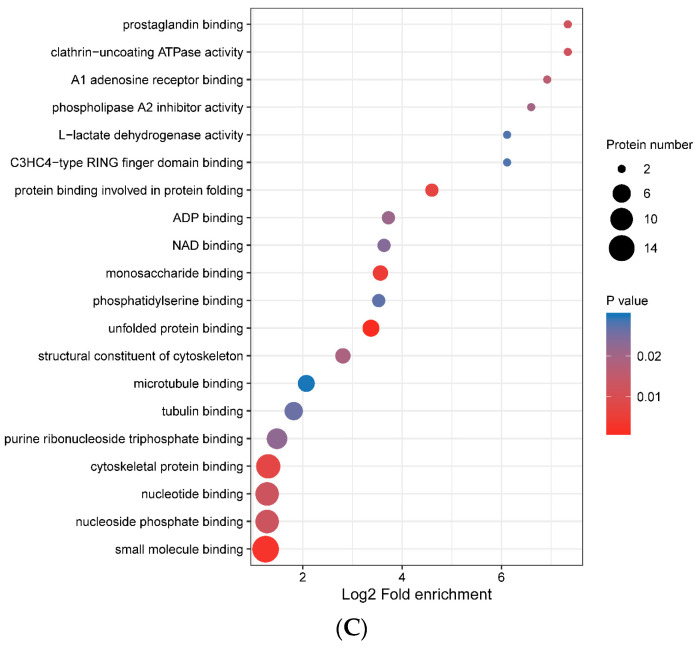
GO enrichment bubble plot of interferon-stimulated gene product 15 (ISG15)-conjugated proteins under different Gene Ontology (GO) categories. (**A**) GO enrichment bubble plot under the GO category of biological process. (**B**) GO enrichment bubble plot under the GO category of cellular component, (**C**) GO enrichment bubble plot under the GO category of molecular function.

To better understand cellular functions and pathways impacted by the porcine ISG15-conjugation system, GO-enrichment analysis results were summarized with respect to GO annotation categories of biological process (Figure 5A), cellular component (Figure 5B) and molecular function (Figure 5C). It is notable that GO-enrichment targets assigned to biological process mainly belonged to pathways associated with nucleotide metabolism, such as ribonucleoside monophosphate metabolic process, ATP metabolic process, ribonucleoside triphosphate metabolic process, ribonucleoside triphosphate biosynthetic process and ribonucleoside diphosphate metabolic process (Figure 5A and Table S4). Although it is unknown whether ISGylation of proteins involved in these processes would accelerate or inhibit nucleotide metabolism, any modulation of nucleotide metabolism would definitely affect virus genome replication, due to its dependence on host nucleotide metabolic machinery. Meanwhile, consistent with GO-enrichment biological process results, GO-enrichment molecular function assignments to ISG15-conjugation substrates identified pathways involved in ADP binding, purine ribonucleoside triphosphate binding, nucleotides binding and nucleoside phosphate binding as well (Figure 5C, Table S4), while also identifying protein binding pathways involved in protein folding and unfolded protein binding (Table S4). Taken together, GO-enrichment analysis results further emphasized that ISGylation in porcine cells mainly modulates host metabolic pathways, especially pathways involved in nucleotide metabolism.

### 3.5. Protein-Protein Interaction Networks

**Figure 6:**
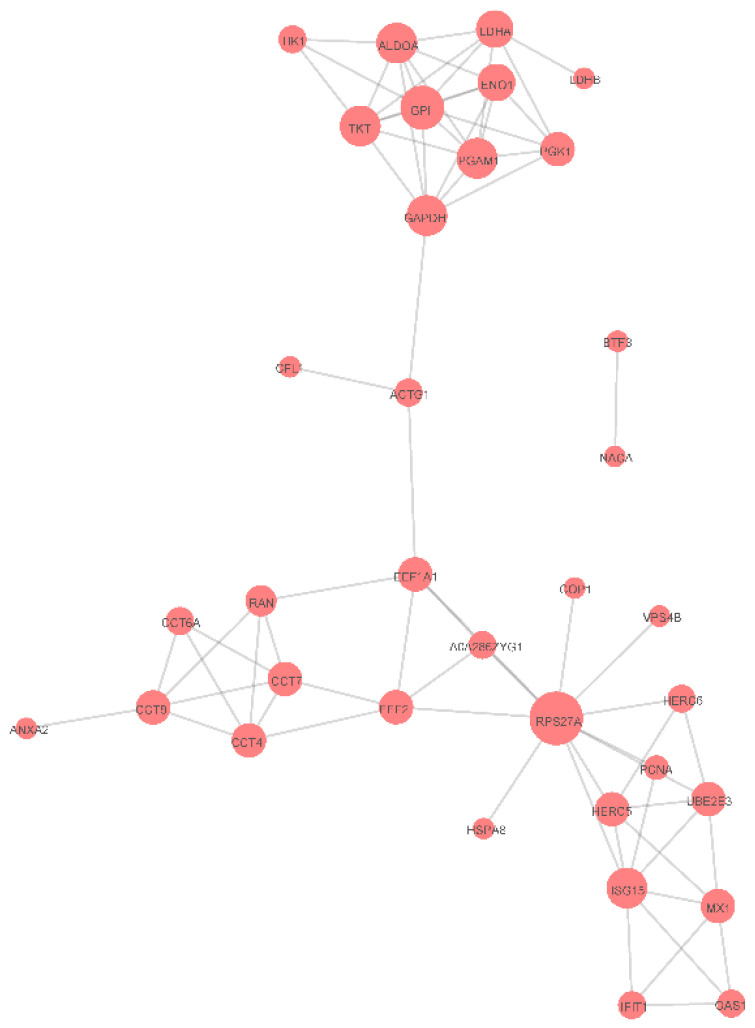
Protein-protein interaction network for identified interferon-stimulated gene product 15 (ISG15)-conjugates in porcine cells.

It has been reported that ISGylation of certain proteins could inhibit protein-protein interactions (PPIs) between ISG15-conjugated proteins and their interacting partners [32,33]. Therefore, to further understand ISGylation roles in porcine cells, a PPI network was constructed based on identified ISGylation candidates (Figure 6). It was notable that ISGylation-associated E3 ligases Herc5 and Herc6, ubiquitin conjugating enzyme E2 E3 (UBE2E3), along with ISG15 itself and other ISGs (MX1, IFIT1 and OAS1) form a cluster of interacting proteins related to functions of ISGs and the ISGylation system. Furthermore, it is also notable that another cluster of metabolism-related genes (GAPDH, GPI, ENO1, LDHA and LDHB) were enriched in the PPI network. However, mechanisms whereby ISGylation impacts PPIs within these clusters of genes and corresponding functions of these PPI networks require further investigation.

## 4. Discussion

Although no universal role has been proposed for ISGylation, ISG15 expression is strongly up-regulated upon IFNs stimulation and likely plays an indispensable role in host innate immunity. Interference with host ISGylation by viral deubiquitinating enzymes has been implicated in virus virulence as well; for example, partial relief of the ISG15-antagonistic function effect of porcine reproductive and respiratory syndrome virus (PRRSV) non-structural protein 2 (NSP 2) ovarian tumor (OTU) protease domain produced a recombinant virus with reduced virulence in vivo [34]. However, little is known regarding the identities of ISG15-conjugated substrates. Results of previous proteomics studies suggest ISG15-conjugated substrates are not only limited in number, but may also be species-specific and lacking of conserved ISGylation motifs [23,30]. Notably, host ISG15 may conjugate to viral proteins during different stages of viral replication in a virus-specific manner [35,36], but no common structural or primary motif associated with ISG15 conjugation to viral protein as well [31]. Conversely, here proteomics analysis detected 191 porcine ISG15-modified peptides in ISG15-Mab-enriched porcine proteins. Notably, subsequent analysis of protein sequences upstream and downstream of putative porcine cell protein ISGylation sites identified no highly conserved motifs, although sequences upstream of ISGylation sites exhibited moderate aa biases toward specific residues.

For the 98 identified porcine cell ISGylation substrate candidates, the top 5 biological process category annotation terms assigned to them included “regulation of biological process”, “cellular metabolic process”, “organic substance metabolic process”, “primary metabolic process” and “nitrogen compound metabolic process”; these assignments aligned with GO-enrichment results as further evidence that nucleotide and glucose metabolic pathways were targeted by the porcine ISG15-conjugation system. Taken together, these data indicate that the porcine ISG15-conjugation system mainly targets host metabolic pathway proteins as targets for ISGylation. Thus, the alteration of host metabolism may serve as the primary mechanism underlying the ISG15 antiviral effect observed in porcine cells.

Besides proteins involved in various metabolic pathways, our data also suggested that some well-defined ISGs, such as MX1, IFIT1, and OAS1, could serve as substrates for the ISG15 conjugation system in porcine cells. These results align with ISGylation results reported for MX1 and other ISGs (e.g., RIG-I, PK-R in human cells) [30], although our data suggest that additional ISGs may be targeted by ISG15. On the one hand, ISG15-mediated ISGylation has been reported to interfere with protein ubiquitination of some lysine residues [37] that may prevent degradation of IRF3, ultimately leading to increased IFN-β expression [38]. It would be interesting to know if ISGylation of MX1, IFIT1, OAS1 antiviral proteins could confer such “gain of function” roles to them as well. On the other hand, ISGylation has also been shown to modulate enzyme activity, since conjugation of ISG15 to protein phosphatase 1B (PPM1B) has been shown to suppress PPM1B activity [39]. Thus, ISGylation of certain antiviral enzymes, such as MX1 and OAS1, may inhibit their enzyme activities as well, although this scenario requires further confirmation. Nevertheless, it is notable that multiple ISGylation sites (K6, K8, K13, K29, K128 and K142) within ISG15 itself were identified. Although poly-ISG15 chains or auto-ISGylation of ISG15 itself have not yet been reported (as previously observed for K68-linked polyubiquitin chains), such scenarios involving the linking together of poly-ISG15 chains are theoretically possible via participation of one or more of the several abovementioned lysine residues of porcine ISG15.

In human and mouse models, protein ISGylation depends on an enzyme cascade that resembles the cascade involved in ubiquitination. Both cascades involve E1-activating enzymes, E2-conjugating enzymes and E3-ligases [40]. Only a limited number of ISG15-specific E3 ligases have been described so far [28,29,41] and include Herc5 (human), Herc6 (mouse), ARIH1 and TRIM25 [41]. Of the porcine ISGylation candidates identified here, notably Herc6 was identified as a potential substrate possessing multiple potential ISGylation sites that could be modified via the porcine ISG15 conjugation system; thus, porcine Herc6 may act as an ISG15-specific E3 ligase, such that ISGylation of Herc6 may potentially regulate its own E3 ligase activity. Moreover, preliminary results (data not shown) detected no pISG15 conjugation to substrates within cells of a human IFNs-stimulated HeLa-pISG15 stable cell line, suggesting that human Herc5 does not function as an E3 ligase that can conjugate porcine ISG15 to substrate. Meanwhile, it has been reported that ubiquitination interferes with ISGylation by blocking protein-protein interactions (PPIs) [20]. Therefore, a PPI network was constructed for ISGylation candidates identified in this work that contained two clusters of interacting proteins. One cluster included ISG15, ISG15-related ISGylation E3 ligases Herc5 and Herc6 and ubiquitin-conjugating enzyme UBE2E3, along with several ISGs (MX1, IFIT1 and OAS1); the other cluster included metabolism-related genes (GAPDH, GPI, ENO1, LDHA and LDHB). Collectively, these results lend further support to our speculation that metabolic pathways and ISGs involved in antiviral activities are natural targets of the porcine ISGylation system. 

## 5. Conclusions

In conclusion, proteomics analysis of porcine ISGylation substrates identified 98 potential protein substrates of pISG15-conjugation. Gene Ontology (GO) annotation and enrichment analyses, along with protein-protein interaction (PPI) network analysis of these proteins, demonstrated that ISGylation occurring within porcine cells mainly impacted host metabolic pathways. Such pathways were especially biased toward nucleotide metabolism, with some ISG-encoded proteins and related proteins also acting as potential ISGylation substrates. These data imply that antiviral functions of porcine ISG15 may rely on modulation of host metabolism and antiviral regulatory loops mediated by some ISGs. Finally, our proteomics analysis results not only identify novel host ISGylation targets, but also provide insights into natural roles of mammalian ISG15 and ISGylation systems.

## Figures and Tables

**Table 1:** Basic statistical table of mass spectrometry (MS) results.

Peptides	Modified Peptides	Identified Proteins	Identified Sites
82281	262	98	190

## Data Availability

The data presented in this study are available on request from the corresponding authors.

## Supplementary Materials

The following are available online at https://www.mdpi.com/2076-393X/9/2/164/s1, Figure S1. Length distribution chart for all peptides identified via mass spectrometry analysis. Table S1, identified peptides sequence putative modification sites and protein description: Table S2, ISG15-conjugated proteins under each GO category; Table S3, COG functional classification of ISG15-conjugated proteins; Table S4, GO-enrichment of involving pathways of ISG15-conjugated proteins.
