# Supplementary figures and images for: Proteomic Analysis of ISGylation in Immortalized Porcine Alveolar Macrophage Cell Lines Induced by Type I Interferon
Source: Vaccines (Basel). 2021 Feb 17;9(2):164. doi: 10.3390/vaccines9020164 (PMC7922875; doi:10.3390/vaccines9020164)

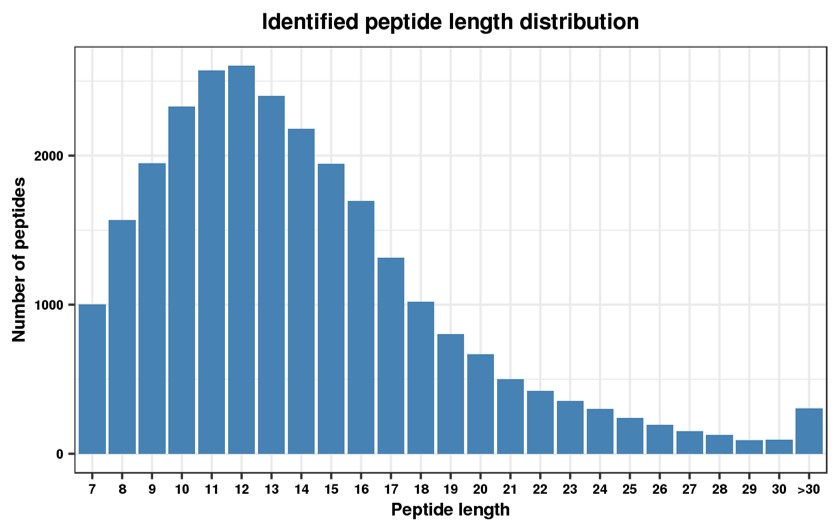

Supplement: Supplementary file 1 [file vaccines-09-00164-s001.zip › Supplementary Figure.1.jpg]
